# Various significant connections of the male pelvic floor muscles with special reference to the anal and urethral sphincter muscles

**DOI:** 10.1007/s12565-019-00521-2

**Published:** 2019-12-23

**Authors:** Janyaruk Suriyut, Satoru Muro, Phichaya Baramee, Masayo Harada, Keiichi Akita

**Affiliations:** Department of Clinical Anatomy, Tokyo Medical and Dental University (TMDU), 1-5-45 Yushima, Bunkyo-ku, Tokyo, 113-8510 Japan

**Keywords:** Anal canal, Levator ani muscle, Pelvic floor muscle, Perineum muscle, Urethra

## Abstract

The male pelvic floor is a complex structure formed by several muscles. The levator ani muscle and the perineal muscles are important components of the pelvic floor. The perineal muscles comprise the external anal sphincter, bulbospongiosus, superficial transverse perineal muscles, and ischiocavernosus. Although the connections of the muscles of the pelvic floor have been reported recently, the anatomical details of each muscle remain unclear. In this study, we examined the male pelvic floor to clarify the connection between the muscles related to function. Fifteen male pelvises were used for microscopic dissection, and three male pelvises were used for histological examination. On the lateral aspect, the perineal muscles were connected to each other. Bundles of the levator ani muscle extended to connect to the perineal muscles. In addition, the extended muscle bundle from the levator ani muscle and the perineal muscles surround the external urethral sphincter. On the medial aspect, the levator ani muscle and the external anal sphincter form the anterior and posterior muscular slings of the anal canal. The connection between the perineal muscles and levator ani muscle indicates a possible close relationship between the functions of the urethra and anus.

## Introduction

The perineum is the caudal layer of the pelvic floor, which is involved in various types of perineal surgeries, such as a perineal prostatectomy. This region is recognized as a particularly complex region in males compared to females (Muro et al. 2018; Nakajima et al. 2017; Oh and Kark 1973). The morphology of the male perineum has been described in general anatomy textbooks (e.g., Standring 2016). The perineum consists of several muscles, known as the perineal muscles, which play a role in controlling the opening of the rectum and urogenital passages (Schuenke et al. 2006; Standring 2016; FCAT 1998). The levator ani muscle is in the deep muscle layer of the perineal muscles, which are essential in supporting the abdominal and pelvic organs. This was confirmed by magnetic resonance imaging and histological studies (Wei and Delancey 2004).

Generally, the perineal muscles are believed to be attached to the perineal body. Previous studies reported that the perineal body is a fibromuscular node located between the urogenital triangle and the anal canal, and is a major point of attachment for the perineal muscles, levator ani muscle, and smooth muscles (Oh and Kark 1973; Zhai et al. 2011; Wu et al. 2015). However, Henle (1866) and Plochocki et al. (2016) found that the perineal body was not a major site of attachment due to a connection between the small muscle fibers in the male perineum area. Recently, Muro et al. (2018, 2019) demonstrated that the perineal body contains both smooth and skeletal muscles; the smooth muscle could be removed from the perineal area to clearly expose the skeletal muscle.

The topography of each perineal muscle has been previously discussed (Matsubara et al. 2003; Salerno et al. 2006; Simunovic et al. 2012). Results from a gross dissection showed that the anterior portion of the levator ani muscle is thick and its fibers run in different directions (Ayoub 1979b). Courtney (1950) demonstrated that the levator ani muscle bundle decussated through the perineal body to form the deep external anal sphincter. Moreover, previous histological studies found that the levator ani muscle is related to the external anal sphincter on the anterolateral portion of the anal canal (Uchimoto et al. 2007; Tsukada et al. 2016). Although the connections among the pelvic floor muscles have been investigated, the connections between the perineal muscles and levator ani muscle in the perineal region have not been described in detail and thus remain unclear.

Hence, we investigated the connection and arrangement of the male pelvic floor muscles through macroscopic and histological examinations to identify the exact relationship between muscle bundles, especially in the region anterior to the anal canal and the region around the membranous part of the urethra.

## Materials and methods

Eighteen Japanese cadavers (age range 62–97 years, mean age 80.9 years) were used for the examinations. The cadavers were fixed in 10% formalin and preserved in 8% alcohol. The cadavers used in the present study were donated to the Department of Anatomy, Tokyo Medical and Dental University, Japan. The format of the document is congruent with the Japanese law entitled Act on Body Donation for Medical and Dental Education. Before their deaths, all donors voluntarily declared that their remains would be donated as materials for educational study. This voluntary donor system of cadavers is applied throughout Japan, and our study completely complies with the current laws in Japan. Cadavers with pathologies or an abnormality that affected the pelvic floor were excluded from the study. The study protocol was approved by the Medical Research Ethics Committee of Tokyo Medical and Dental University, Japan (No. M2018-006).

In the dissection under stereo microscope examination, 17 halves of the pelvises (10 cadavers) were used for dissection from the lateral aspect. The skin, vessels, nerves, and connective tissues were removed to examine the pelvic floor muscles. After dissecting the pelvic floor muscle from the lateral aspect, six halves of the pelvises were randomly chosen. Thereafter, the internal organs, vessels, and nerves were removed to examine the pelvic floor muscles from the medial aspect. Moreover, five pelvises (5 cadavers) were used for dissection from the inferior aspect. After removing the skin and vessels, the pelvic floor muscles were observed from the inferior aspect. Subsequently, the bulbospongiosus was cut along the midline, and the bulbs of the penis were carefully removed to examine the directions of the muscle from the inferior aspect.

Histological examinations were performed on three pelvises (3 cadavers). The pelvic outlet, which includes the distal tip of the prostate, membranous urethra, and the proximal part of the crus and bulb of the penis, and the pelvic floor muscle surrounding the membranous urethra were obtained en bloc. The tissues were embedded in paraffin and transverse serial sections were cut 5-μm thick at intervals of 1 mm, and stained with Elastica-van Gieson.

## Results

### Lateral aspect

**Fig. 1:**
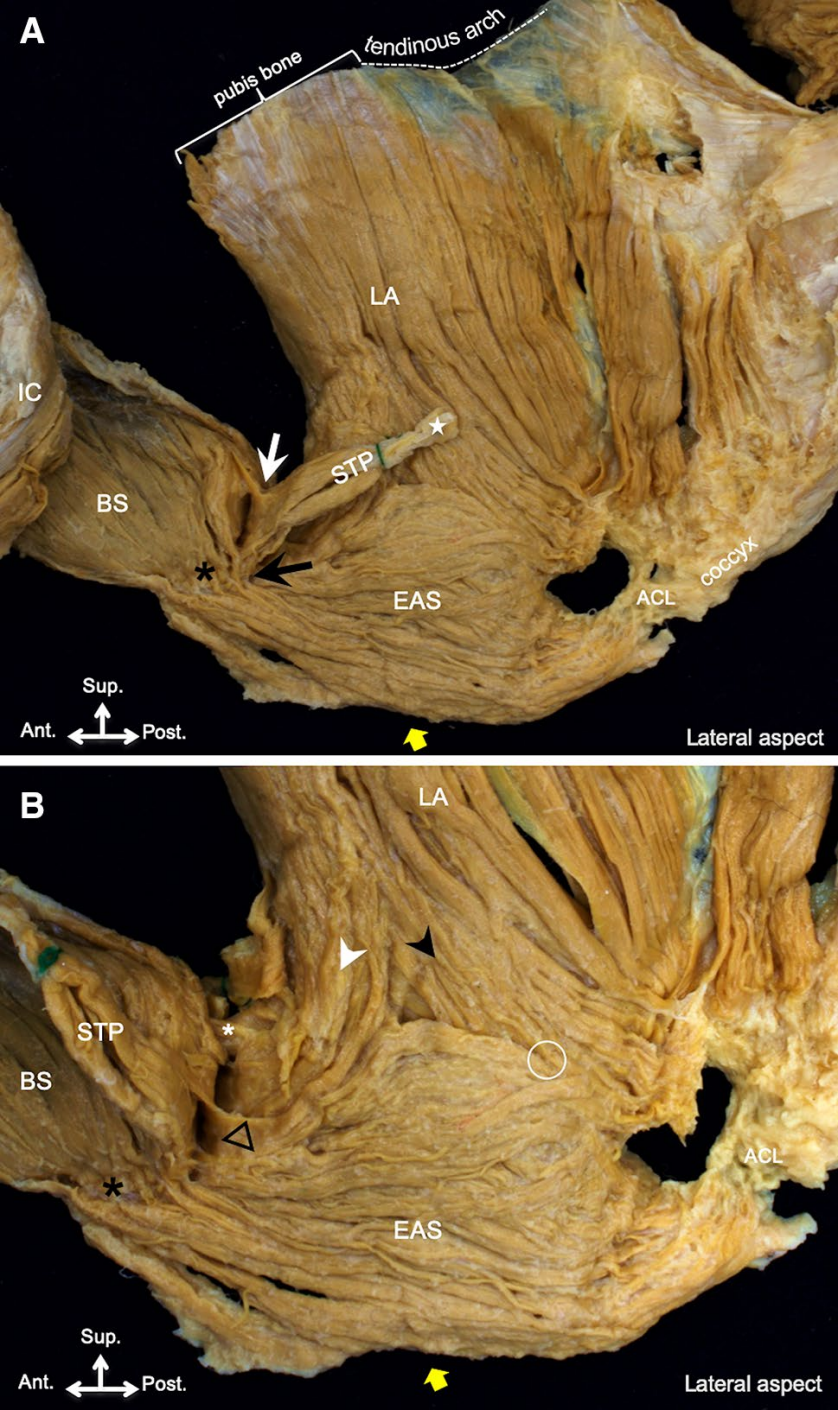
Pelvic floor muscle. **a** Pelvic floor muscle on the lateral aspect, which is composed of the IC, BS, STP, EAS, and LA. The star represents the lateral tendon of the STP; white and black arrows, the connection among the STP, BS, and EAS; black asterisk, the connection between the BS and EAS. **b** After the lateral reflections of the STP, the white and black arrowheads represent the anterior and posterior muscle bundle of the LA, respectively; white asterisk, the connection between the LA and BS; black triangle, the connection between the STP and LA; circle, the blending point between the LA and EAS. *ACL* anococcygeal ligament, *BS* bulbospongiosus, *EAS* external anal sphincter, *IC* ischiocavernosus, *LA* levator ani muscle, *STP* superficial transverse perineal; yellow arrow, anal opening

On the lateral aspect, the outer surfaces of the pelvic floor muscles were observed after connective tissue removal. The bulbospongiosus, superficial transverse perineal, external anal sphincter, and levator ani muscles were identified; however, the borders between these muscles and attachment sites in the central region were not clearly observed (Fig. 1).

In all specimens, the muscle bundles of the bulbospongiosus covering the outer surface of the penile bulb and the corpus spongiosum were connected to the contralateral muscle bundle at the median line (Fig. 1). In addition, the bulbospongiosus surrounded the membranous part of the urethra and was attached to the lateral surface of the corpus cavernosum. The superficial transverse perineal originated from the ischial tuberosity, which ran inferomedially to connect to the external anal sphincter (indicated by a black arrow in Fig. 1a). The muscle also extended anteriorly to connect to the lateral surface of the bulbospongiosus (indicated by a white arrow in Fig. 1a) and extended superiorly to connect to the lateral surface of the levator ani muscle (indicated by a black triangle in Fig. 1b).

The external anal sphincter was a single circular skeletal muscle surrounding the anal canal, which could not be completely identified as different parts. In all specimens, some parts of the muscle bundles of the anal sphincter muscle extended anteriorly and posteriorly. At the anterior side of the anal canal, the inferior 2/3 of the external anal sphincter extended to connect to the inferomedial surface of the bulbospongiosus (indicated by a black asterisk in Fig. 1a, b). At the posterior side of the anal canal, the inferior 2/3 of the muscle converged posteriorly and extended superoposteriorly to attach to the posterior surface of the coccyx via the anococcygeal ligament, which was described in detail by Muro et al. (2014) (Fig. 1a).

**Fig. 2:**
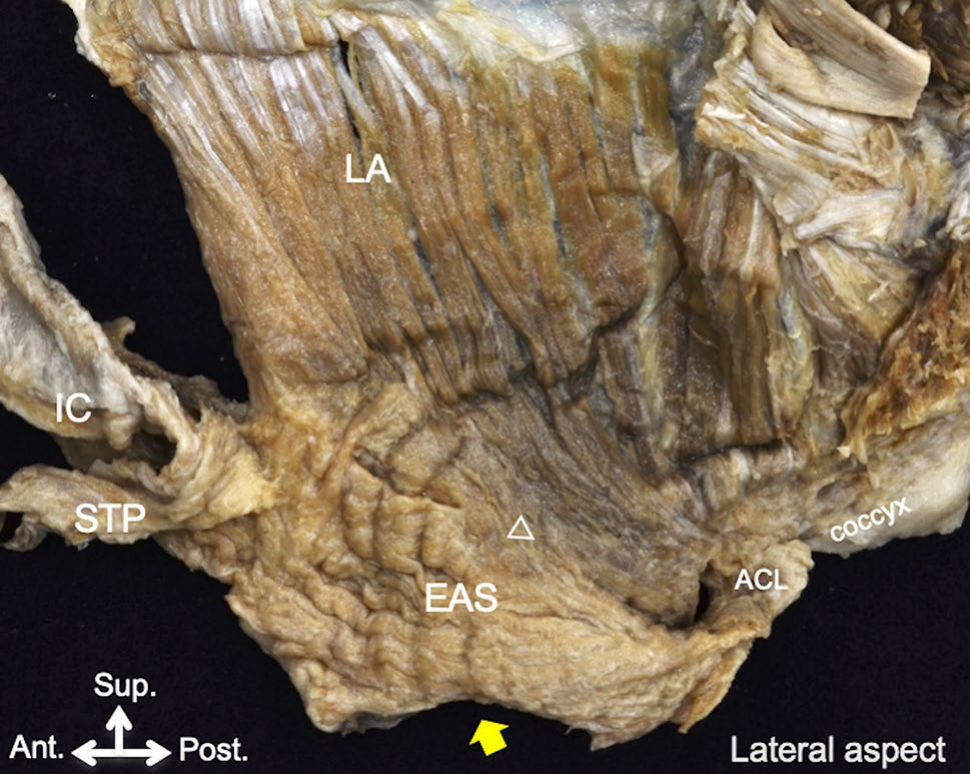
The muscle bundle of the LA extended to the coccyx via the ACL (white triangle). *ACL* anococcygeal ligament, *EAS* external anal sphincter, *IC* ischiocavernosus, *LA* levator ani muscle, *STP* superficial transverse perineal; yellow arrow, anal opening

The levator ani muscle originated from the inner surfaces of the body and superior ramus of the pubis and from the tendinous arch of the levator ani muscle. The part of the levator ani muscle that originated from the inner surface of the body of the pubis was divided into the anterior and posterior muscle bundles in 10 of the 17 specimens (indicated by white and black arrowheads, respectively, in Fig. 1b). The anterior and posterior muscle bundles extended inferoanteriorly and inferoposteriorly, respectively, running along with and on the external anal circular muscle (Fig. 1b). The anterior muscle bundle usually covered the outer surface of the external anal sphincter, and in 41% (7 of 17) of the specimens, the extended muscle bundles connected to the bulbospongiosus at the superior surface (indicated by a white asterisk in Fig. 1b). However, in 41% (7 of 17) of the specimens, the anterior muscle bundles could not be clearly identified on the lateral aspect. The posterior muscle bundle usually ran on the inner surface of the external anal sphincter and was connected to the superior one-third of the muscle on the posterior wall of the anal canal (indicated by a white circle in Fig. 1b). In the remaining 18% (3 of 17) of the specimens, a part of the posterior muscle bundle also ran on the outer surface of the external anal sphincter and extended to the coccyx via the anococcygeal ligament, connecting to the inferior 2/3 of the external anal sphincter (indicated by a triangle in Fig. 2).

**Fig. 3:**
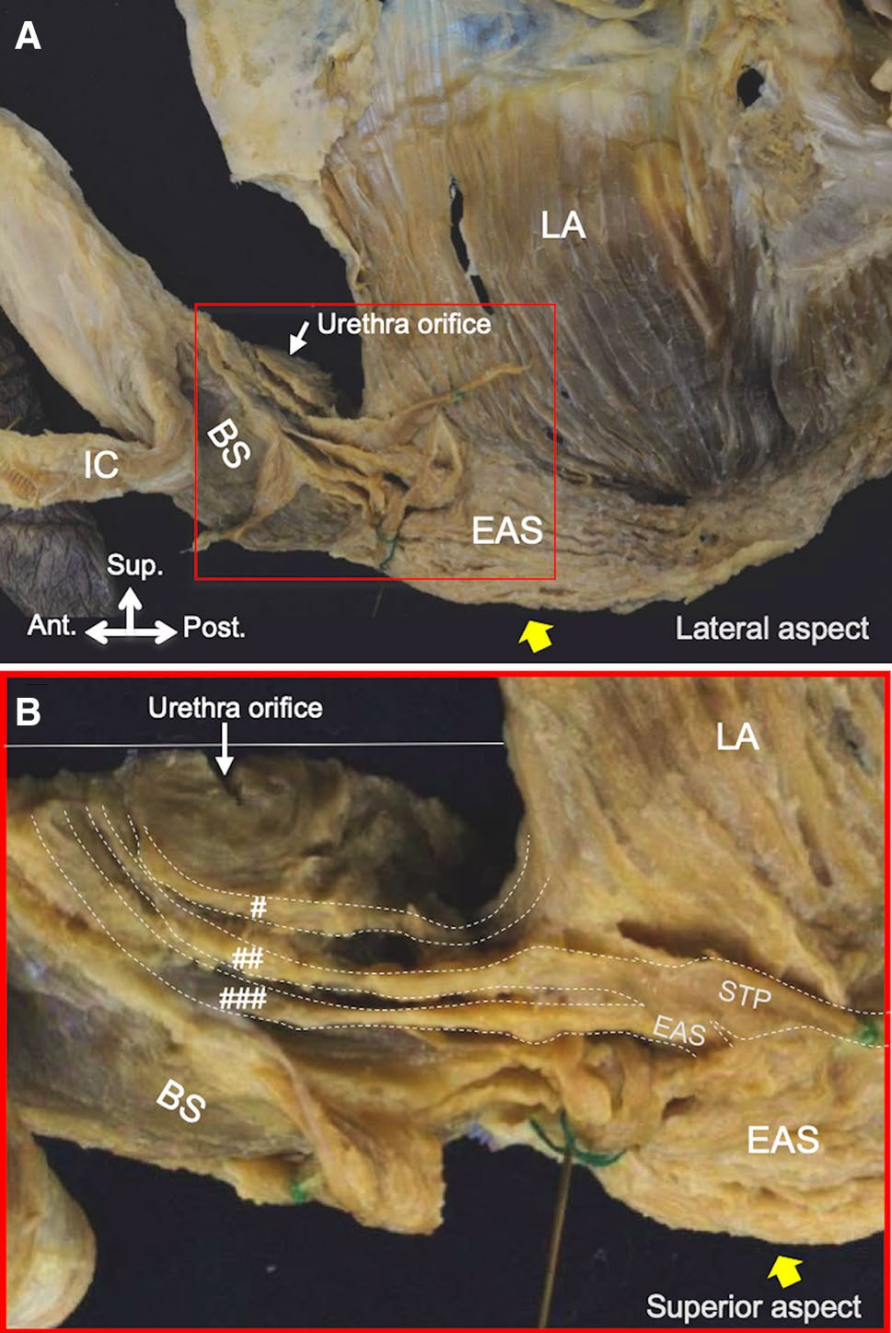
The pelvic floor muscle related to the internal urethral orifice on the lateral aspect. **a** The red box indicates the area shown in **b**. **b** The muscle bundle of the LA, STP, and EAS extending anteriorly to the BS and surrounding the internal urethral orifice from the superior aspect. *BS* bulbospongiosus, *EAS* external anal sphincter, *IC* ischiocavernosus, *LA* levator ani muscle, *STP* superficial transverse perineal; white line, midline of the pelvic floor; yellow arrow, anal opening; #, anterior extending muscle bundles of the LA; ##, anterior extending muscle bundles of the STP; ###, anterior extending muscle bundles of the EAS

In 24% (4 of 17) of the specimens, parts of the muscle bundles from the levator ani muscle, superficial transverse perineal, and external anal sphincter ran anteriorly along with the bulbospongiosus to surround the urethra (Fig. 3).

### Medial aspect

**Fig. 4:**
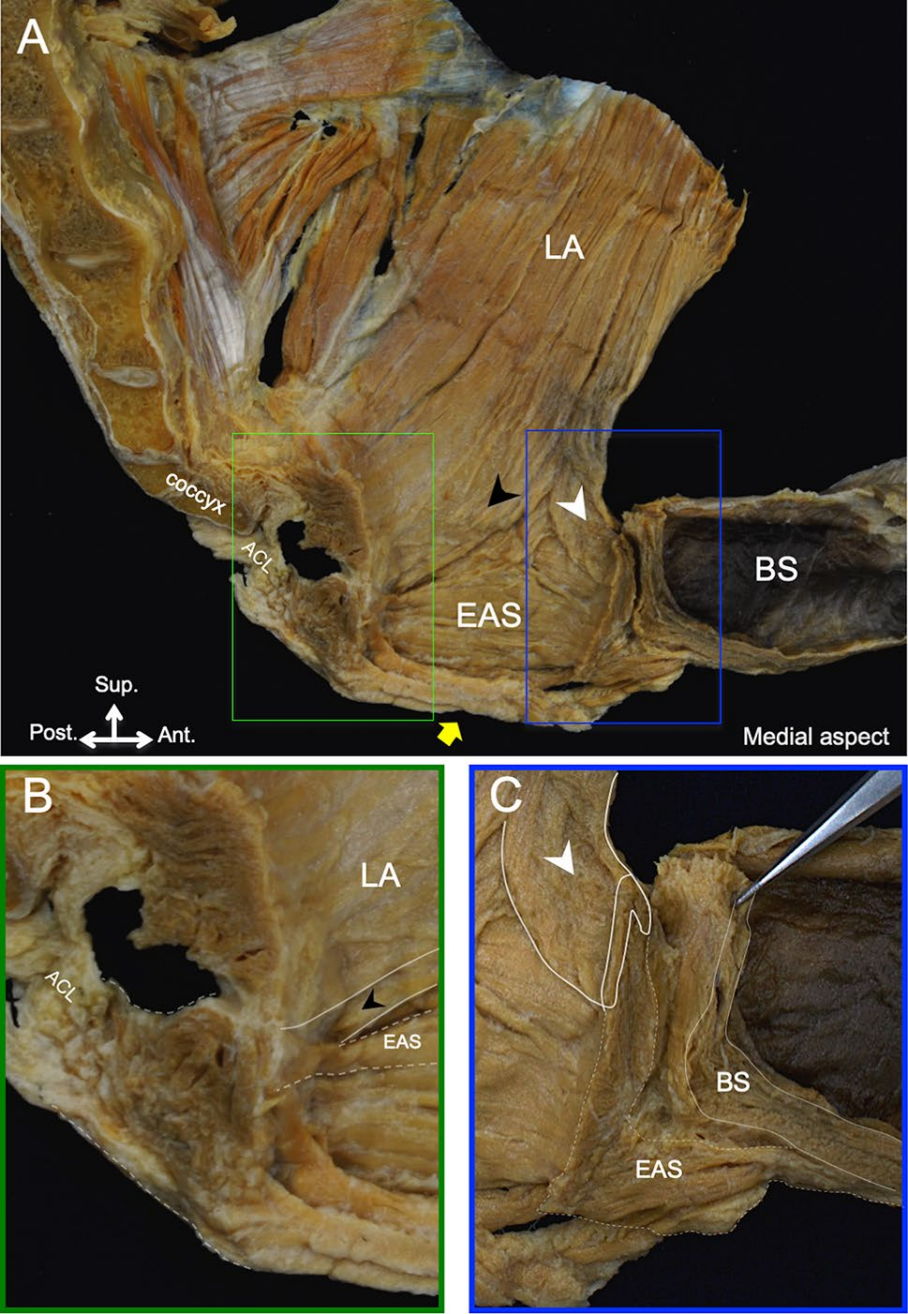
The connection among the pelvic floor muscles on the medial aspect. **a** The green and blue boxes indicate the areas shown in B and C. **b** Formation of the posterior muscular sling on the posterior portion of the anal canal. **c** Formation of the anterior muscular sling on the anterior portion of the anal canal. *ACL* anococcygeal ligament, *BS* bulbospongiosus, *EAS* external anal sphincter, *LA* levator ani muscle; black arrowhead, posterior muscle bundle of the levator ani muscle; white arrowhead, anterior muscle bundle of the levator ani muscle; yellow arrow, anal opening

On the medial aspect, the inner surfaces of the muscles of the pelvic floor were observed after the removal of the internal organs, vessels, and nerves in six specimens (Fig. 4a). The posterior muscle bundle of the levator ani muscle ran posteroinferiorly, and the cut ends of the muscle bundle were observed on the medial surface (Fig. 4b). These cut ends were considered to be the regions attached to the longitudinal smooth muscle of the rectum, as described by Muro et al. (2014) and Tsukada et al. (2016). In the posterior portion of the muscles surrounding the anal canal, the posterior muscle bundle of the levator ani muscle ran on the surface of the superior one-third of the external sphincter muscle (Fig. 4b). The posterior muscle bundle was connected to the contralateral muscle bundle to form the posterior muscular sling (Fig. 4b).

In the anterior portion of the muscles surrounding the anal canal, the anterior muscle bundle of the levator ani muscle ran approximately one-third of the way along the superior and inner surfaces of the external sphincter muscle (Fig. 4c). The anterior muscle bundle was connected to the contralateral muscle bundle to form the anterior muscular sling (Fig. 4c). On the median plane, the inferior one-third of the external anal sphincter extended superoanteriorly to attach to the inferomedial surface of the bulbospongiosus (Fig. 4c). Between the external anal sphincter and the bulbospongiosus, a small gap was noted (Fig. 4c). This small gap was surrounded by various muscular connections observed from the lateral aspect and was occupied by some tissues, including adipose tissue.

### Inferior aspect

**Fig. 5:**
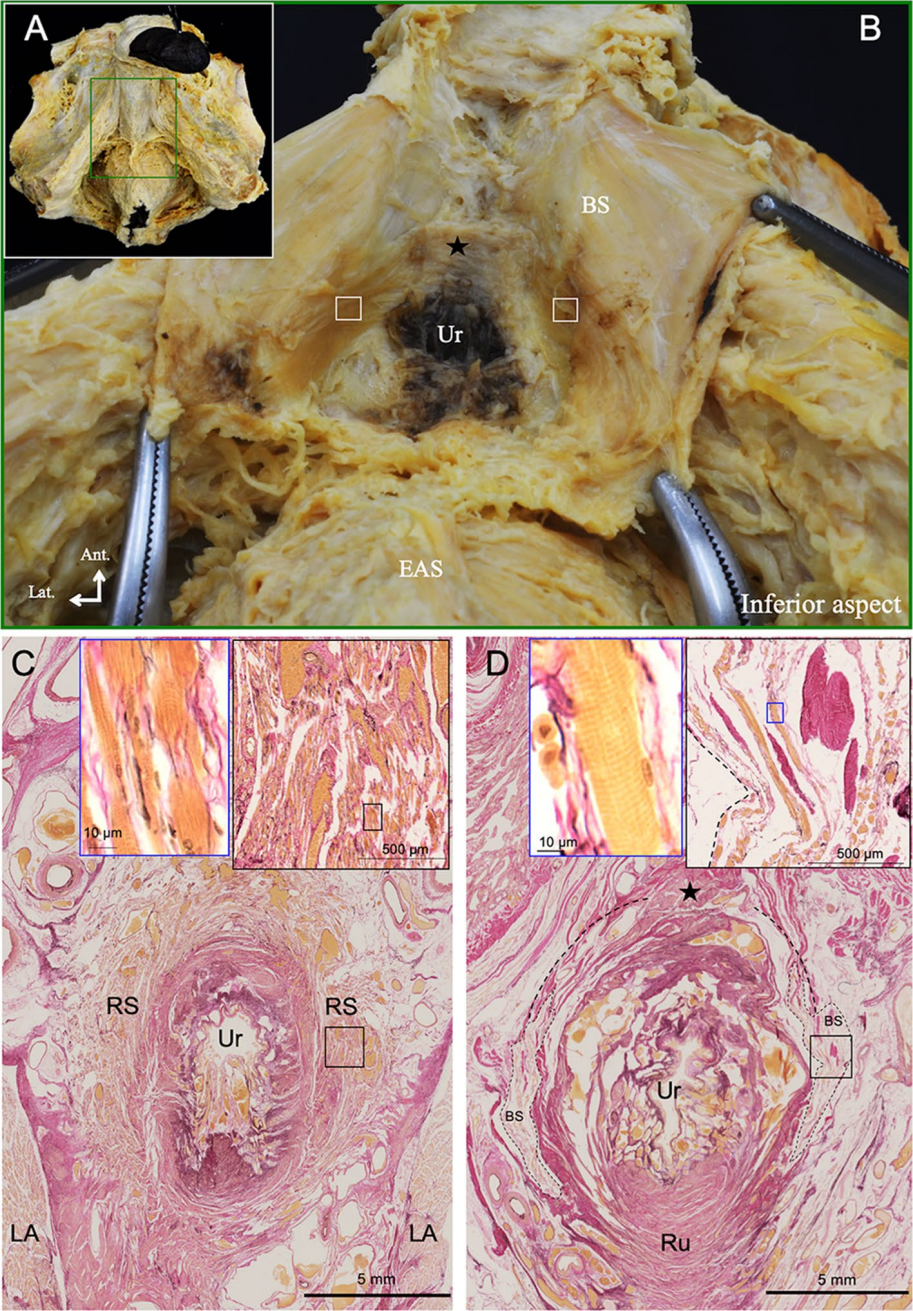
The inferior aspect of the BS. **a** The inferior aspect of the BS. **b** The magnified region indicated by the green square in **a**; the inner surfaces of the BS are shown. The membranous part of the urethra was surrounded by the muscle bundles of the BS. **c** Histological sections of the membranous urethra in the superior region stained by Elastica-Van Gieson stain. The black and blue squares indicate the RS and skeletal muscle cells of RS, respectively. **d** Histological sections of the membranous urethra in the inferior region stained by Elastica-Van Gieson stain. The black and blue squares indicate the BS and skeletal muscle cells of BS, respectively. The white squares and black dotted lines indicate the muscle bundle of the BS surrounding the internal urethral orifice. The black star indicates the tendinous fibers. Bulbospongiosus. *EAS* external anal sphincter, *LA* levator ani muscle, *RS* rhabdosphincter, *Ru* rectourethralis, *Ur* membranous part of the urethra

The bulbospongiosus was opened laterally to the left and right after cutting on the median line in five specimens, and the corpus spongiosum and the bulb of the penis were removed (Fig. 5a, b). The inner surfaces of the bulbospongiosus surrounded the membranous part of the urethra anterolaterally (indicated by a square in Fig. 5b). Both sides of the muscle bundles of the bulbospongiosus continued to the tendinous fibers, and the fibers were connected to each other (indicated by a square and dotted line in Fig. 5b, d). The axial histological sections of the membranous part of the urethra and the surrounding region were observed. In the section of the more superior region of the membranous part, the urethra was surrounded by the rhabdosphincter and was sandwiched by the levator ani muscle (Fig. 5c). In the more inferior region of the membranous part, the urethra was not observed to be surrounded by the rhabdosphincter, but was surrounded by the bulbospongiosus (Fig. 5d). The urethra was attached to the rectourethralis muscle posteriorly.

## Discussion

**Fig. 6:**
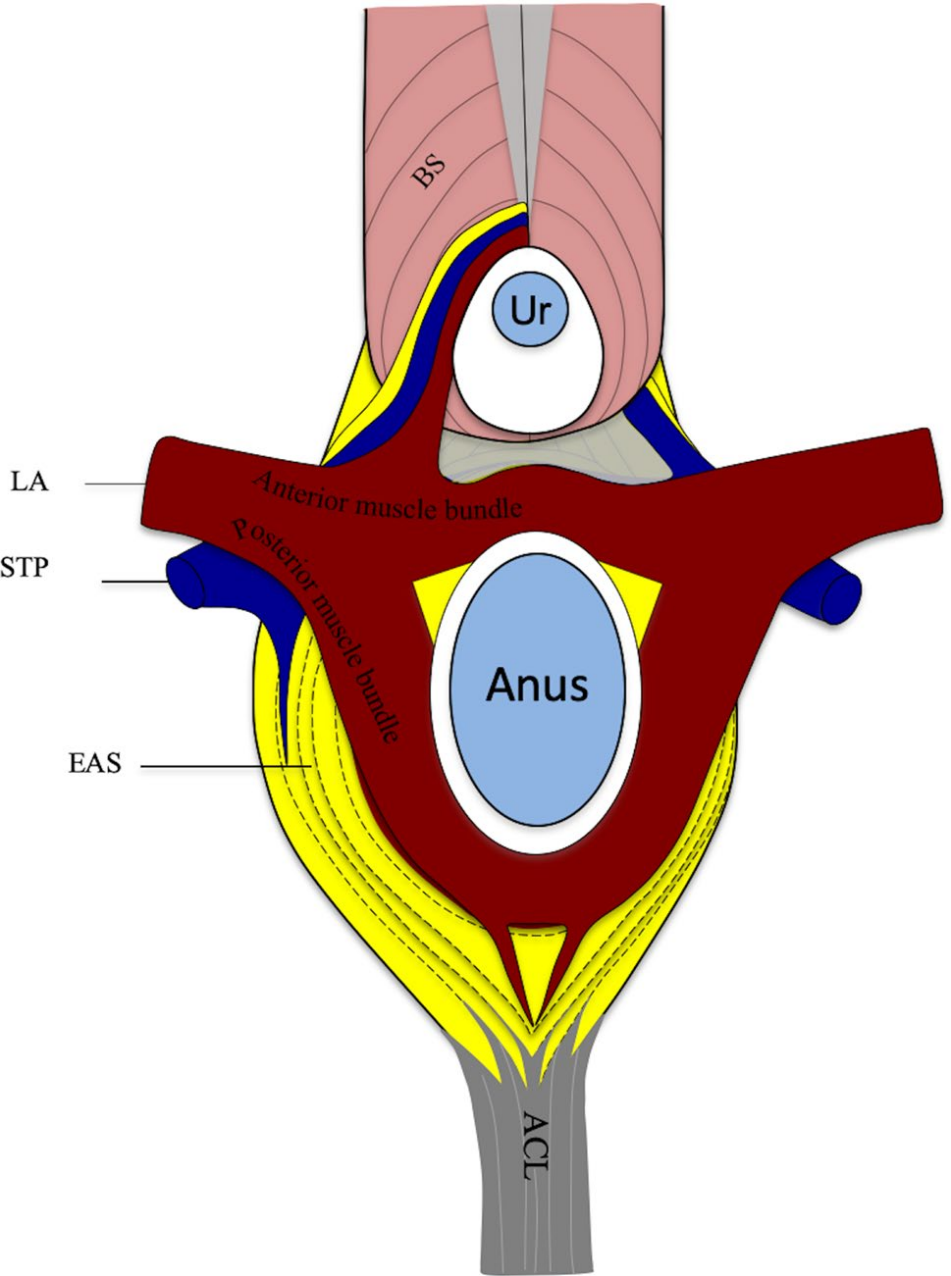
Muscle connections and arrangement of the pelvic floor muscle. The illustration shows the connection of the muscle on the anterior, anterolateral, and posterior portions of the anal canal on the superior aspect. *ACL* anococcygeal ligament, *BS* bulbospongiosus, *EAS* external anal sphincter, *LA* levator ani muscle, *STP* superficial transverse perineal, *Ur* membranous part of the urethra

In the present study, we found various muscle connections among the perineal muscles that have not been clearly described previously. Some of these muscle connections may be closely related to the urethral sphincter. The various connections of the perineal muscles and the muscle bundles of the levator ani muscle, based on the anatomical findings in the superior aspect, are shown in Fig. 6. The superficial transverse perineal is connected to the external anal sphincter and the bulbospongiosus. The external anal sphincter is connected to the bulbospongiosus. The muscle bundles of the levator ani muscle are divided into the anterior and posterior muscle bundles, and these muscle bundles surround the anal canal that is superior to the external anal sphincter. In addition, in some specimens, the extending muscle bundles from the external anal sphincter, the superficial transverse perineal, and the anterior muscle bundle of the levator ani muscle run around the membranous part of the urethra together with the bulbospongiosus.

### Perineal muscles

Studies by Oh and Kark (1972) and Stoker (2009) identified the superior, middle, and inferior parts of the external anal sphincter. However, our study found that the external anal sphincter was a single muscle group that could not be differentiated into different parts. Previous reports showed that the external anal sphincter extends anteriorly and inserts into the perineal body (the central tendon of the perineum) on the anterior portion of the anal canal (Oh and Kark 1972; Ayoub 1979a; Stoker 2009). However, we found that the external anal sphincter extends anteriorly to connect to the bulbospongiosus instead and thus does not insert into the perineal body. Moreover, in the posterior portion of the anal canal, three parts of the external anal sphincter were attached to the coccyx by the anococcygeal ligament. This finding was in agreement with that of Ayoub (1979a) and Muro et al. (2014).

Generally, the perineal muscles in males have been described as independent muscles that insert into the perineal body. A previous study reported that the small muscle bundle of the external anal sphincter connects to the bulbospongiosus (Peikert et al., 2015). This result was supported by the study of Akarawa et al. (2010), which reported the development of the external anal sphincter in midterm fetuses by serial sections. Moreover, their study found that the superficial or deep external anal sphincter was formed by the muscle fibers from the bulbospongiosus anlage. In addition, the study by Henle (1866) indicated that the perineal muscles are connected by tiny muscle fibers, while Plochocki et al. (2016) found that the muscles in the male perineal area are connected to each other like a single sheet. Previous studies have presented little connection between the perineal muscles (Henle 1866; Plochocki et al. 2016). However, in our study, dissection revealed that the perineal muscle bundles are not only strongly connected to each other in various ways, but also have a connection with the levator ani muscle.

### Perineal body

The perineal body has been described as a fibromuscular tissue and as the central point of attachment for several structures, including the perineal muscles, the levator ani muscle, longitudinal muscle of the rectum, and the rectourethralis (Oh and Kark 1973; Zhai et al. 2011; Wu et al. 2015). Using magnetic resonance imaging, Larson et al. (2010) confirmed the presence of the perineal body and demonstrated that it has three regions, which was supported by the studies by Nakajima et al. (2017) and Muro et al. (2018, 2019). They reported that the perineal body in both males and females is found in the area between the rectum and urogenital structures and contains both smooth and skeletal muscles. However, in our study, we observed only the connection among the skeletal muscles after removing the smooth muscle and connective tissue. The smooth connective tissue and the connecting skeletal muscles could be generally referred to as the perineal body.

### Levator ani muscle

The levator ani muscle has been described as a broad muscular sheet of variable thickness, arising from the body and superior ramus of the pubis and from the tendinous arch of the levator ani muscle. The levator ani muscle is subdivided into three portions based on the origin and insertion: the pubococcygeus, iliococcygeus, and puborectalis. However, a well-known textbook indicated that the boundaries among each part of the levator ani muscle could not be completely identified (Standring 2016). A previous study showed that the muscle bundles of the levator ani muscle are attached to the coccyx to form the pelvic floor muscle (Stoker 2009). Ayoub (1979b) found that the levator ani muscle consists of anterior and posterior portions; the muscle of the anterior portion is a thick bundle, which originates from the pubis and runs in several directions, and the posterior portion is thin with parallel muscle bundles that originate from the tendinous arch. In our study, we investigated the outer and inner aspects and confirmed the different terminations of the levator ani muscle. Dissection revealed that the anterior muscle bundle of the levator ani muscle covered the outer surface of the external anal sphincter, which correlates with the results of the study of Courtney (1950). In addition, the anterior muscle bundle also connected to the superficial transverse perineal and the bulbospongiosus, which has never been reported in any previous study. The posterior muscle bundle of the levator ani muscle was related to the external anal sphincter on the lateral portion of the anal canal and was adjoined to the external anal sphincter on the posterior portion of the anal canal. Our findings were consistent with those of Uchimoto et al. (2007) and Tsukada et al. (2016).

### External urethral sphincter and its supporting system

According to the studies of Oelrich (1980) and Strasser et al. (1996), the area around the rhabdosphincter is composed of striated muscle fibers extending from the neck of the bladder to the perineal membrane. The rhabdosphincter, or external urethral sphincter, which is located in the membranous urethra at the apex of the prostate, was described as omega-shaped (Strasser et al. 1996) or inverted horseshoe-shaped (Murakami et al. 2002). A previous study also found that the rhabdosphincter is sandwiched between both sides of the levator ani muscle and is connected to the inferomedial margin of the levator ani muscle by a thick fascia (Matsubara et al. 2003; Hinata and Murakami 2014). Moreover, it was reported that the posterior portion of the rhabdosphincter is attached to the rectourethralis (Murakami et al. 2002; Soga et al. 2008) and that the inferior portion of the rhabdosphincter lies on the perineal membrane (Hinata and Murakami 2014). In our study, we found extra muscle bundles surrounding the membranous part of the urethra. The extra muscle bundles usually consisted of extending the muscle bundles from the bulbospongiosus. In addition, the extra muscle bundles also consisted of muscle bundles from the levator ani muscle, the superficial transverse perineal, and the external anal sphincter. It has been generally considered that the rhabdosphincter functions to compress the membranous urethra (and the other skeletal muscle bundles surrounding the area play a role in supporting the rhabdosphincter muscle) and that the external urethral sphincter is the upper circular element that surrounds the urethra in the apex of the prostate in males (Standring 2016). However, our study showed that the external urethral sphincter could be a complex system of striated muscles, such as the rhabdosphincter, the muscle bundles of the bulbospongiosus, and the extra muscle bundles from the superficial transverse perineal, and the anterior muscle bundle of the levator ani muscle. Therefore, a close relationship between the sphincter functions of the urethra and the anus is possible.
